# Development and validation of next generation sequencing based 35-gene hereditary cancer panel

**DOI:** 10.1186/s13053-020-00141-2

**Published:** 2020-04-28

**Authors:** Wing Chan, Mianne Lee, Zhen Xuan Yeo, Dingge Ying, Keith A. Grimaldi, Craig Pickering, Michael M. S. Yang, Senthil K. Sundaram, Lawrence C. H. Tzang

**Affiliations:** 1 Prenetics Limited, 7/F, Prosperity Millennia Plaza, 663 King’s Road, Quarry Bay, Hong Kong SAR, China; 2 Exercise and Nutritional Genomics Research Centre, DNAfit Ltd, FORA, 71 Central Street, London, EC1V 8AB UK; 3 Department of Biomedical Science, City University of Hong Kong, 1A-107, 1/F, Block 1, To Yuen Building, Kowloon Tong, Hong Kong SAR, China

**Keywords:** Hereditary cancer, Next generation sequencing, Multigene panel testing, Analytical validation, Genetic testing

## Abstract

**Background:**

Understanding the genetic basis of cancer risk is a major international endeavor. The emergence of next-generation sequencing (NGS) in late 2000’s has further accelerated the discovery of many cancer susceptibility genes. The use of targeted NGS-based multigene testing panels to provide comprehensive analysis of cancer susceptible genes has proven to be a viable option, with the accurate and robust detection of a wide range of clinically relevant variants in the targeted genes being crucial.

**Methods:**

We have developed and validated a targeted NGS-based test for hereditary cancer risk assessment using Illumina’s NGS platform by analyzing the protein-coding regions of 35 hereditary cancer genes with a bioinformatics pipeline that utilizes standard practices in the field. This 35-gene hereditary cancer panel is designed to identify germline cancer-causing mutations for 8 different cancers: breast, ovarian, prostate, uterine, colorectal, pancreatic, stomach cancers and melanoma. The panel was validated using well-characterized DNA specimens [NIGMS Human Genetic Cell Repository], where DNA had been extracted using blood of individuals whose genetic variants had been previously characterized by the 1000 Genome Project and the Coriell Catalog.

**Results:**

The 35-gene hereditary cancer panel shows high sensitivity (99.9%) and specificity (100%) across 4820 variants including single nucleotide variants (SNVs) and small insertions and deletions (indel; up to 25 bp). The reproducibility and repeatability are 99.8 and 100%, respectively.

**Conclusions:**

The use of targeted NGS-based multigene testing panels to provide comprehensive analysis of cancer susceptible genes has been considered a viable option. In the present study, we developed and validated a 35-gene panel for testing 8 common cancers using next-generation sequencing (NGS). The performance of our hereditary cancer panel is assessed across a board range of variants in the 35 genes to support clinical use.

## Background

Understanding the genetic basis of cancer risk is a major international endeavor that began in 1980s. The identification of germline mutations within *BRCA1* and *BRCA2,* two genes associated with breast cancer, in the 1990s was a major milestone in the research of hereditary cancer. The emergence of next-generation sequencing (NGS) in late 2000’s has further accelerated the discovery of many cancer susceptibility genes. Currently, over 100 cancer susceptibility genes that are associated with cancer risk have now been discovered [1], with more genes are being continually identified [2].

While the discovery of such new cancer genes is a critical first step, additional clinical studies are needed to independently validate these discoveries. However, the development of such clinical evidence for the new genes typically lags behind their discoveries. Based on our assessment of the strength of available clinical evidence, we have generated a hereditary cancer panel including 35 genes for 8 hereditary cancer types (breast cancer, ovarian cancer, uterine cancer, colorectal cancer, melanoma, pancreatic cancer, stomach cancer and prostate cancer). Early and reliable detection of pathogenic germline variants in susceptibility genes for the 8 cancer types will enable prediction of cancer risks in order to prevent or delay the onset of these hereditary cancers, with the design of personalized cancer risk management plans.

The present study is designed to validate this targeted 35-gene NGS cancer panel. While NGS has shown to be quite useful in assessing hereditary cancer risk, it is also recognized that this is a complex technology, requiring significant validation efforts. In particular, several variables related to laboratory procedures and bioinformatics pipeline can influence the accuracy of these results. Furthermore, despite the technology being available for about a decade, there is still significant uncertainty about the impact of different variables on the test accuracy. This implies that analytical and functional validation studies of new targeted NGS panels are critically important.

We describe the development and validation of next generation sequencing-based 35-gene hereditary cancer panel (Prenetics Limited) through the detection of single nucleotide variants and small insertions/deletions in 35 genes related to 8 hereditary cancers described above. Our validation strategy is governed by the guidelines for NGS from the American College of Medical genetics and Genomics (ACMG) [3] and the College of American Pathologist (CAP). In the present study, our validation is designed to provide a good representation of possible variant types across the 35 genes in our cancer panel. The studies included well-characterized DNA specimens, where DNA had been extracted using blood of individuals whose genetic variants had been previously characterized by the 1000 Genome Project [4, 5] and the Coriell Catalog.

## Methods

### Hereditary Cancer panel

The hereditary cancer panel (Prenetics Limited) is designed to assess clinically relevant germline mutations in 35 genes associated with hereditary risk for breast, ovarian, colorectal, pancreatic, prostate, uterine, stomach cancers and melanoma through the detection of single nucleotide variants (SNVs) and small insertions/deletions (Indel; up to 15-25 bp) located in the DNA coding sequences, nearby flanking regions (20 bp flanking of each exon) and known splice regions in the targeted genes. The gene-cancer associations are summarized in Table S1. These genes include *APC, ATM, BAP1, BARD1, BMPR1A, BRCA1, BRCA2, BRIP1, CDH1, CDK4, CDKN2A, CHEK2, EPCAM, GREM1, MEN1, MITF, MLH1, MRE11A, MSH2, MSH6, MUTYH, NBN, PALB2, PMS1, PMS2, POLD1, POLE, PTEN, RAD50, RAD51C, RAD51D, SMAD4, STK11, TP53,* and *XRCC2* (Table S2). This panel of genes was generated based on our assessment of the strength of available clinical evidence, including the following criteria: a) Testing of the cancer genes as recommended by professional guidelines (e.g., from National Comprehensive Cancer Network (NCCN) [6, 7], American Society of Clinical Oncology (ASCO) [8]), b) Genes found to be valid cancer genes by systematic reviews, c) Genes in which pathogenic mutations have been reported by multiple research studies or reputed resources such as Clingen. With continuous gene discoveries and clinical validation, additional genes or variants may be included to our panel to expand the covered conditions in the future.

### Library preparation and next-generation sequencing

The genomic DNA (gDNA; 25–500 ng) was diluted with 10 mM Tris buffer and sheared using Covaris S2 sonicator to achieve target peak of 100 to 350 bp following the standard SonoLab 7 settings (Covaris, Woburn, MA). The genomic libraries were prepared following the KAPA Hyper Preparation kit standard protocol (Roche, Pleasanton CA). All individual DNA libraries were validated both quantitatively and qualitatively by using a fluorescence-based measurement method and an automatic gel electrophoresis method respectively. Successfully validated DNA libraries were pooled and hybridized to custom probes by using Nimblegen EZ choice enrichment kit (Roche, Pleasanton CA), in which the in-house designed biotinylated-labelled oligonucleotide probes were synthesized to bind to targeted-gene regions according to the human genome reference sequence version GRCh37 (hg19). Target-captured library was prepared according to the manufacturer’s protocol following the SeqCap EZ library preparation guide (NimbleGen, Madison, WI) without modification. Fourteen cycles of PCR were completed for amplification of the captured library. The target enriched DNA library was validated both quantitatively and qualitatively using method described before. In addition, real-time quantitative PCR, as described in the Illumina Sequencing Library real-time quantitative PCR quantification guide (Illumina), was performed to determine the final size-adjusted concentration of the target enriched DNA library. The target-enriched DNA fragments were sequenced by Illumina MiSeq via pair-end, 2 × 150 base pair reads, with an average coverage of at least 50x to ensure data quality. Each sequencing run included one fully characterized positive control. The panel is not intended to detect the following types of variations: somatic mutations, gross rearrangements and deep intronic variation, Alu element insertions, certain homopolymers such as those in PMS1 and other unknown abnormalities. The pattern of variant types varies with the genes and this panel detects a high but variable percentage of known and unknown variants of the classes stated. The 35 genes are assessed for variants within all coding exons. Novel sequence changes in the promoter regions and other non-coding regions will not be detected.

### Quality control (QC)

QC procedures were in-place along the sequencing process to ensure sample identification, high quality of DNA isolation, library preparation, target capture and sequencing. In addition, mapping and variant QC metrics were computed on the sequencing output and used to exclude and re-run failed samples. The QC metrics criteria are detailed in Table S3.

### Bioinformatics processing

Standard bioinformatics analysis pipeline was used to analyze sequencing data with reference to genome GRCh37 (hg19) for sequence alignment. The pipeline includes BWA alignment (0.7.15) and The Genome Analysis Toolkit (GATK 4.0) best practices. Low quality and duplicated reads were removed and followed by Burrows-Wheeler Aligner (BWA 0.7.15) and variant calling based on GATK (4.0) best practices. Variant annotation is performed by using the ANNOVAR Version: 2017-07-17 01:17:05–0400 (Monday, 17 July 2017) [9]. Snakemake [10], a workflow management framework, were implemented to control the execution of the pipeline.

### Variant interpretation

Standardized gene variant nomenclature recommended by the Human Genome Variation Society (HGVS) is adopted. Variants within the reportable range are classified by following the standards and guidelines for sequence variants interpretation of the American College of Medical genetics and Genomics (ACMG) [11] and best practice guidelines for Variant Classification of the Association for Clinical Genomics Science (ACGS) [12]. Variants are classified into five categories, including pathogenic, likely pathogenic, variant of uncertain significance (VUS), likely benign, and benign based on ACMG five-tier classification system [11].

### Validation samples

Two sets of reference materials, obtained from the NIGMS Human Genetic Cell Repository at the Coriell Institute for Medical Research, were used for validation of the 35-gene hereditary cancer panel: (a) Cohort I: 94 genomic DNA reference materials with known SNVs and indels. These samples are selected based on the availability of high-quality variant calls and recommendations by the National Institute of Standards and Technology (NIST) [13] (Table S5**)**. The samples are provided in a blinded manner from The Croucher Laboratory for Human Genomics (CLHG), The Chinese University of Hong Kong to Prenetics. Results are submitted to CLHG for comparing variants calls to 1000 Genome Project and Illumina Platinum Genomes [4, 5]. This allowed us to test the accuracy of the 35-gene Hereditary Cancer Panel in the absence of any prior knowledge of the status or genetic variants in the sample set. Low-confidence variants in 1000 genomes project were independently confirmed by Sanger sequencing. (b) Cohort II: 53 genomic DNA reference materials with positive cancer history and known pathogenic/likely pathogenic variants in *APC*, *ATM*, *BRCA1*, *BRCA2*, *CDKN2A*, *MEN1* and *PTEN* (Table S6). Interpretation of variant calls in these reference materials were compared to the Coriell Catalog. Pathogenic and likely pathogenic variants identified undergo additional confirmatory test using Sanger sequencing.

### Reproducibility and repeatability

In addition to establishing the analytical and functional performances of the 35-gene Hereditary Cancer Panel, inter-run reproducibility and intra-run repeatability were also determined. Inter-run reproducibility was assessed by comparing results of 26 unique reference samples across three distinct batches of sequencing run which are handled by three different laboratory technicians. Intra-run repeatability was computed by comparing the results of reference sample in 20 replicates within the same batch of sequencing run.

### Statistical analysis

The validation studies evaluated the following test performances of our 35-gene hereditary cancer panel: Accuracy = (TP + TN) / (TP + FP + TN + FN); Sensitivity = TP / (TP + FN); Specificity = TN / (TN + FP); where TP - true positives, TN - true negatives, FP - false positives, and FN - false negatives. Inter-run reproducibility and intra-run repeatability were calculated as the ratio of concordant calls to total calls. The corresponding confidence intervals (CIs) were calculated using R package: binom (V1.1–1) by the method of bootstrapping regression models [14].

## Results

**Fig. 1:**
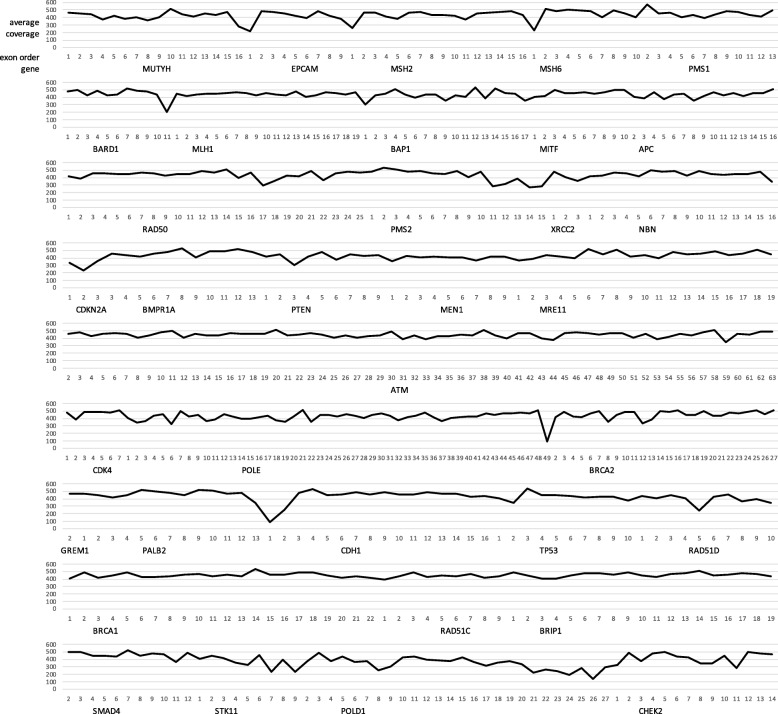
Exonic coverage of 35 hereditary cancer genes based on a benchmark sequencing run with 43 samples

In the present study, we developed and validated a 35-gene panel for testing 8 common cancers using next-generation sequencing (NGS). This capture-based next generation sequencing panel detects single nucleotide variants (SNVs) and small insertions/deletions (Indel; up to 15-25 bp) located in the DNA coding sequences, nearby flanking regions (20 bp flanking of each exon) and known splice regions in the targeted genes. Based on a benchmark sequencing run with 43 samples, we assessed the exonic coverage in the targeted regions (Fig. 1 and Table S4). The average depth of coverage for 35 genes is at least 400x, ranging from 87.8x to 574.1x across a total of 548 exons. Using 4820 variants from the reference materials obtained from the NIGMS Human Genetic Cell Repository, we found a sensitivity of 99.9% and a specificity of 100% (Table 3) across 94 samples in the blinded group (Cohort I) and 53 samples in the positive reference group (Cohort II). In addition, the inter-run reproducibility and intra-run repeatability are 99.8 and 100%, respectively.

### Analytical validation

SNV and indel detection were examined on Cohort I and Cohort II for a total of 147 reference samples. Descriptive statistics of variants, stratified by variant type, for the reference materials is provided in Table 1. Each of the discordant sites was independently confirmed by Sanger sequencing supporting of our panel’s calls as true positives or true negatives. Out of the 4820 total variants validated, 6 were discordant between our panel and the 1000 Genome Project and Illumina Platinum Genomes [4, 5, 15]. These potential false negatives, i.e. variants identified by the 1000 Genomes Project but not by our panel, are located in the pseudogene region of *PMS2* (Chr7:601315). All the 6 false negatives correspond to the same benign variant in 6 different samples. The analytical validation results of our 35-gene hereditary cancer panel is summarized in Table 2 and Table 3.

**Table 1:** Descriptive statistics of variants from reference materials

**Specimen**	**Number of variants**		
	**SNVs**	**Indels**	**Total**
Cohort I: blinded group (*n* = 94)	4738	20	4758
**Specimen**	**Number of pathogenic/likely pathogenic variants**		
	**SNVs**	**Indels**	**Total**
Cohort II: positive reference samples (*n* = 53)	31	31	62
**TOTAL**	4769	51	4820

**Table 2:** Overview of results for 4820 variants, of which 62 variants had been classified as pathogenic or likely pathogenic in *APC, ATM, BRCA1, BRCA2, CDKN2A, MEN1* and *PTEN*

Gene	Total Variants	True Positives	False Positives	False Negatives
APC	622	622	0	0
ATM	176	176	0	0
BAP1	17	17	0	0
BARD1	298	298	0	0
BMPR1A	82	82	0	0
BRCA1	399	399	0	0
BRCA2	577	577	0	0
BRIP1	331	331	0	0
CDH1	116	116	0	0
CDK4	0	0	0	0
CDKN2A	29	29	0	0
CHEK2	19	19	0	0
EPCAM	111	111	0	0
GREM1	31	31	0	0
MEN1	252	252	0	0
MITF	4	4	0	0
MLH1	34	34	0	0
MRE11	140	140	0	0
MSH2	104	104	0	0
MSH6	104	104	0	0
MUTYH	76	76	0	0
NBN	260	260	0	0
PALB2	41	41	0	0
PMS1	10	10	0	0
PMS2	362	362	0	6^a^
POLD1	124	124	0	0
POLE	322	322	0	0
PTEN	7	7	0	0
RAD50	10	10	0	0
RAD51C	10	10	0	0
RAD51D	55	55	0	0
SMAD4	0	0	0	0
STK11	8	8	0	0
TP53	85	85	0	0
XRCC2	4	4	0	0
**TOTAL**	**4820**	**4820**	**0**	**6**^a^

^a^6 variants at the position Chr7:6013153 in PMS2 is a false negative. But, this is in a pseudogene region and is also a benign variant

**Table 3:** Assessment of Prenetics hereditary cancer panel test performance in detection and interpretation of single nucleotide variants and small insertions/deletions

Specimen	Total Variants	True Positives	False Positives	False Negatives	Sensitivity	Specificity
Cohort I: blinded group (*n* = 94)	4758	4758	0	6	99.9%	100%^a^
Cohort II: positive reference samples (*n* = 53)	62	62	0	0	100.0%	100%^a^
**TOTAL**	**4820**	**4820**	**0**	**6**	99.9%	100%^a^

^a^no false positive was identified

### Functional validation

Functional validation does not refer to independent experimental validation of these variants but refers to the combination of analytical accuracy and interpretation accuracy of functional assessment, i.e. whether a variant was accurately classified as positive or negative based on ACMG five-tier classification system [12]. Positive results include pathogenic and likely pathogenic variants, whereas negative results include benign, likely benign variants as well as variant of uncertain significance (VUS). To measure the functional accuracy, sensitivity and specificity, Cohort II of 53 genomic DNA reference materials with positive cancer history and known pathogenic/likely pathogenic variants in *APC*, *ATM*, *BRCA1*, *BRCA2*, *CDKN2A*, *MEN1* and *PTEN* were sequenced. Positive (pathogenic and likely pathogenic) variants identified undergo additional confirmatory test using Sanger sequencing. Interpretation of variant calls in these reference materials were compared to the Coriell Catalog. An overview of the functional variants, stratified by variant type, is provided in Table 1. A total of 62 pathogenic and likely pathogenic variants were identified with no false positives or false negatives (Table 2 and Table 3).

### Reproducibility and repeatability

Procedures for calculating reproducibility and repeatability are described in the methods section. We found that the concordance between replicates of the intra and inter-run replicates was > 99.9%. The inter-run reproducibility was 99.8% (95% CI: [99.6, 99.9%]) over 7998 of 8018 variants while intra-run repeatability was 100% (95% CI:[99.9, 100%]) over 6060 variants. These measurements were calculated based on all detected variants.

## Discussion

Within the past decade, sequencing technology has developed rapidly with the adoption and leverage of high-throughput next-generation sequencing. Along with the advances in bioinformatics analysis, the use of targeted NGS-based multigene testing panels to provide comprehensive analysis of cancer susceptible genes has proven to be a viable option, in comparison to traditional germline testing for mutation in a single gene approach [3, 16–22]. The performances of these panels depend on various factors including but not limited to target enrichment platform, sequencing technology, bioinformatics pipeline, and variant interpretation. Accurate and robust detection of a wide range of clinically relevant variants in the targeted genes is critical [6, 7].

To address these challenges, we have developed and validated a 35-gene hereditary cancer panel using Illumina’s NGS platform. This panel is designed to sequence and analyze the protein-coding regions of the targeted genes for the identification of cancer-causing mutations in 8 different cancers: breast, ovarian, prostate, uterine, colorectal, pancreatic, stomach cancers and melanoma. The aim of the present validation study was to assess the analytical and functional performances of the 35-gene hereditary cancer panel. No false positive was identified in the analytical validation study and no discordance was observed in the functional validation study. In addition, the functional validation cohort consists of samples with clinically significant variants as well as those with technically challenging variants. These technically challenging variants include complex indels (delins) and long indels (> 10 bps) (detailed in Table S7). Delins could be reported as multiple distinct neighboring variants and it would require careful evaluation for accurate interpretation. On the other hand, alignments of reads with long indels tend to have higher error rate. In the present validation study, neither false positive nor false negative was identified among these technically challenging variants. High sensitivity, specificity, reproducibility and repeatability were observed across both SNVs and indels including pathogenic/likely pathogenic variants and technically challenging variants. The superior performance reported here, particularly in the functional validation study with samples of affected individuals, underlines the importance of an accurate variant interpretation process.

Our panel does not analyze the following types of variations: somatic mutations, gross rearrangements and deep intronic variation, Alu element insertions, certain homopolymers such as those in PMS1. Similarly, our panel does not detect novel sequence changes in the promoter regions and other non-coding regions (except for variants with 20 base pair flanking regions that would include splicing regions). However, since virtually all of the known cancer pathogenic variants are within coding or splicing regions, we believe that this would not be a significant limitation of this validation study.

Until recently, hereditary cancer testing has been focused on the identification of point mutations or small indels with high analytical sensitivity and specificity. However, the rate of detection of mutations in these genes rarely reached the expected high frequency even in the family cancer clinical setting. Undetected mutations in families with mutilple cases of the disease in successive generations might be explained by alternative mechanism of gene inactivation, namely large genomic rearrangements (LGR). Though not routinely detectable, LGRs including deletions and duplications of multiple exons is responsible for a variable but significant proportion of hereditary cancer mutations, particularly in *BRCA* genes [23]. LGRs of *BRCA1* may account for up to one-third of all disease-causing alterations in various populations, while LGRs in *BRCA2* less frequently observed [24]. The rapid evolution of NGS techologies and bioinformatics workflow may allow the search for small variants and LGRs with a single platform, providing high accuracy for clinical decisions.

## Conclusions

In conclusion, we have developed and validated the 35-genes hereditary cancer which is designed to detect clinically relevant germline mutations associated with hereditary risk for common cancer types (i.e. breast, ovarian, colorectal, pancreatic, prostate, uterine, stomach cancers and melanoma). The performance of the test is assessed across a board range of variants in the 35 genes to support clinical use. The present validation study confirmed high sensitivity (99.9%) and specificity (100%) across a total of 4820 variants. The reproducibility and repeatability are 99.8 and 100%, respectively.

## Supplementary information

**Additional file 1: Table S1.** Known associations between genes in the 35-gene hereditary cancer panel and caner type. **Table S2.** Prenetics hereditary cancer panel reportable range. **Table S3.** Quality control metrics. **Table S4.** Exonic coverage of 35 hereditary cancer genes based on a benchmark sequencing run with 43 samples. **Table S5.** Summary of Coriell samples (1000 Genome) used for assessment of SNV and indel detection. **Table S6.** Summary of 53 Coriell samples used for assessment of SNV and indel detection and interpretation. **Table S7.** Technically challenging variants in the present validation.

## Data Availability

Details of the reference samples selected for the present validation can be found: https://www.coriell.org/0/Sections/BrowseCatalog/.

## Abbreviations

ACMG: American College of Medical genetics and Genomics
ASCO: American Society of Clinical Oncology
ACGS: Association for Clinical Genomics Science
bp: Base pair
BWA: Burrows-Wheeler Aligner
CAP: College of American Pathologist
CIs: Confidence intervals
DNA: Deoxyribonucleic acid
FN: False negatives
FP: False positives
GATK: Genome Analysis Toolkit
gDNA: Genomic DNA
HGVS: Human Genome Variation Society
NCCN: National Comprehensive Cancer Network
NIST: National Institute of Standards and Technology
NGS: Next-generation sequencing
SNVs: Single nucleotide variants
Indels: Small insertions and deletions
TN: True negatives
TP: True positives
VUS: Variant of uncertain significance
